# *In-Silico* Modeling of Tumor Spheroid Formation and Growth

**DOI:** 10.3390/mi12070749

**Published:** 2021-06-25

**Authors:** Meitham Amereh, Roderick Edwards, Mohsen Akbari, Ben Nadler

**Affiliations:** 1 Laboratory for Innovations in MicroEngineering (LiME), Department of Mechanical Engineering, University of Victoria, Victoria, BC V8W 2Y2, Canada; mamereh@uvic.ca; 2 Centre for Advanced Materials and Related Technologies (CAMTEC), University of Victoria, Victoria, BC V8W 2Y2, Canada; 3 Department of Mathematics and Statistics, University of Victoria, Victoria, BC V8W 2Y2, Canada; edwards@uvic.ca; 4 Biotechnology Center, Silesian University of Technology, Akademicka 2A, 44-100 Gliwice, Poland; 5 Department of Mechanical Engineering, University of Victoria, Victoria, BC V8W 2Y2, Canada; bnadler@uvic.ca

**Keywords:** tumor formation, reaction–diffusion equation, human glioblastoma cancer cells

## Abstract

Mathematical modeling has significant potential for understanding of biological models of cancer and to accelerate the progress in cross-disciplinary approaches of cancer treatment. In mathematical biology, solid tumor spheroids are often studied as preliminary *in vitro* models of avascular tumors. The size of spheroids and their cell number are easy to track, making them a simple *in vitro* model to investigate tumor behavior, quantitatively. The growth of solid tumors is comprised of three main stages: transient formation, monotonic growth and a plateau phase. The last two stages are extensively studied. However, the initial transient formation phase is typically missing from the literature. This stage is important in the early dynamics of growth, formation of clonal sub-populations, etc. In the current work, this transient formation is modeled by a reaction–diffusion partial differential equation (PDE) for cell concentration, coupled with an ordinary differential equation (ODE) for the spheroid radius. Analytical and numerical solutions of the coupled equations were obtained for the change in the radius of tumor spheroids over time. Human glioblastoma (hGB) cancer cells (U251 and U87) were spheroid cultured to validate the model prediction. Results of this study provide insight into the mechanism of development of solid tumors at their early stage of formation.

## 1. Introduction

Understanding tumors has been recognized as one the most challenging problems in biology and medicine. Their behavior involves complicated molecular biology and correspondingly complicated dynamics. Although tremendous effort has been devoted to developing therapeutic approaches for tumor suppression, there is still a significant need for new insights into complex aspects of tumors such as genetic instabilities, therapeutic resistance, inter- and intra-tumor heterogeneity, etc. Mathematical modeling is one of the most powerful approaches in predicting different aspects of tumor progression. It can provide quantitative prediction of biological processes and help interpret complicated physiological interactions in the tumor microenvironment. Provided collaboration with experimentalists, mathematical modeling can generate practical mechanistic solutions. Mathematical oncology, for example, promises to allow quantitative determination of effective individual therapies [1]. Therefore, the cross-disciplinary approach of combining mathematical and biological models of cancer has great potential to enhance cancer treatment.

Primary malignant tumors originate from small numbers of cells which become highly proliferative through mutations occurring in oncogenes, tumor suppressors or DNA repair genes [2]. Although the genetic mutations eventually lead to formation of large and complex vascular tumors, they all go through an avascular (devoid of blood vessels) mode at their early stage of growth [3]. Having insight into this stage of growth is valuable in understanding the behavior of tumors at subsequent phases. Tumor spheroids are predominantly used in *in vitro* models of avascular tumor growth [4,5,6,7,8]. They are spherical aggregations of cancer cells which are supplemented with a controlled amount of nutrient concentration. Spheroid size, cell numbers, and fractions of hypoxic, quiescent and proliferative cells are easy to track, which enables researchers to quantitatively investigate the effect of various parameters on the tumors’ behavior [9,10,11].

Over recent decades, avascular tumor spheroids have been extensively studied in the field of mathematical biology [12,13,14,15,16]. In particular, the growth of tumor spheroids is mathematically modeled from different perspectives, e.g., continuum, discrete and hybrid (continuum-discrete) [17].

In the continuum approach, the variables are assumed to be continuous, and described by means of PDEs that incorporate growth and kinetic interaction between components, and diffusion for transport phenomena [18,19,20]. For instance, Burton developed one of the early mathematical models to determine the radius of tumor spheroids by analysis of oxygen distribution inside the tumor [21]. The model could generate a growth curve, which was fitted well by a Gompertzian function. As another example of studies that examined the oxygen dynamics of spheroids, Grimes et al. demonstrated that the growth of spheroids can be obtained using two key parameters: rates of oxygen consumption and proliferation [22]. Greenspan established a model focusing on the growth-inhibiting effect of chemicals produced by disintegrated dead cells at the core of the tumor [23]. He concluded that the growth pattern strongly depends on a balance between the inhibitory effect of chemical components and the proliferation of living cells. Byrne and Chaplain introduced a model that provides the growth of tumor spheroids in response to local nutrient concentration [24]. They assumed that the tumor is an incompressible fluid in which the inside motion is induced by local proliferation of cells. They also incorporated cell-cell adhesion by the Gibbs-Thomson relation, which maintains the tumor’s compactness. They further studied asymmetric perturbations to predict the modes of instability of radially symmetric growth. Further studies considered the effects of pH and the level of oxygen and nutrient as key parameters for the tumor growth rate [25,26]. Anisotropic growth of avascular tumor spheroids was also modeled in the context of continuum mechanics in references [27,28,29,30,31]. These studies modeled the tumor as a hyperelastic material and used multiplicative decomposition of the deformation gradient to investigate spatial distribution of stresses. They highlight the role of mechanical stress on the growth of tumors. Additional continuum-based studies of solid tumor growth are reviewed in references [32,33,34].

On the other hand, discrete approaches can capture the effects of cellular response, signaling pathways, inter- and intra-cellular interactions and tumor microenvironment on the growth of solid tumors [17]. For example, the dynamics of avascular tumor growth is presented using a lattice Monte Carlo model in [35]. The model predicts the growth of spheroids under various nutrient supply conditions. Hybrid models are another approach to modeling the growth of tumors that combine both continuum and discrete approaches to allow descriptions of macroscopic environmental variables (such as nutrient concentration) as well as discrete biological interactions. In the case of solid tumor growth, the clinical-size morphology of tumors can be studied in hybrid models while the cellular pathways and subcellular interactions are also involved [36]. For more details about discrete and hybrid approaches, the reader is encouraged to refer to the following papers [33,37,38,39].

Development of biological patterns, such as animal coat markings and evolution of the enteric nervous system (ENS), is another type of process that often involves cellular proliferation and reaction/diffusion of materials [40,41,42]. To describe such processes, the evolution of a domain boundary is incorporated into the mathematical modeling [43,44,45,46]. This boundary evolution of patterns is very similar to that of tumors, i.e., growth, from a mathematical point of view. In ENS, for instance, the motion of neural precursor cells is studied by solving a PDE describing migrating cells [47,48,49]. Another example is the study done by Simpson to derive an exact solution for a linear reaction–diffusion PDE on a uniformly growing domain [50]. The method was verified by comparing the solution with a numerical approximation. Although they successfully obtained the exact solution for the density function in a growing domain, their solution is restricted to one-dimensional linear and exponential domain growth in Cartesian coordinates, whereas in tumor growth the evolution of a tumor’s boundary is not prescribed and is obtained as the solution in spherical coordinates.

Despite the rich literature on mathematical modeling of solid tumor growth, the initial stage of tumor formation is either neglected or not comprehensively modeled. At this stage, the interplay between adhesive force, due to cell-cell interactions, and repulsive force, due to local pressure gradient, defines the dynamic formation of tumor spheroids, which further affects the subsequent growth phases. To better understand the involved mechanisms, there is still a need to conduct more collaborative research involving biological and mathematical modeling approaches.

Here, we focus on the early phase of tumor spheroid formation, which is mostly missing from previous studies. In this initial phase of spheroid formation, the cell-cell adhesion forces are dominant and drag cells toward each other to make a compact aggregation. This causes the spheroid to shrink. Once cells start to proliferate, the raised concentration of cells within the spheroid produces a pressure which compensates for the adhesion forces. At this point, the balance between forces stops spheroids from shrinking and leads to monotonic growth. This phase carries on until necrosis occurs where the competition between mitosis and necrosis defines the growth pattern. This early transient behavior of cells in tumor spheroid formation is modeled by a reaction–diffusion equation coupled with an ODE. The effect of adhesion forces between cells are incorporated into the system by prescribing a constant boundary condition. This fixed concentration, the so-called relaxed concentration, implicitly models an equilibrium condition at which adhesion forces and intrinsic pressure are in balance.

Both analytical and numerical solutions were obtained for the change in the radius of a tumor spheroid at the early stage of formation. The theoretical model was validated against the formation and growth of tumor spheroids generated from glioblastoma cell lines.

## 2. Model Formulation

A tumor spheroid is considered to be a system of particles (cells) with continuous change in their properties such as concentration, velocity, etc. To start modeling this system, the material time derivative of the tumor spheroid’s mass is written as a balance equation for the continuum concentration of cells within the system:

(1) $$\frac{\partial m}{\partial t}=\frac{\partial }{\partial t}\int_{V}C\left(x,t\right)dV=\int_{V}\Pi \;dV\;,$$

where *m* is the mass of the tumor, $C\left(x,t\right)$ is local concentration of cells, x is the position vector, Π is a volumetric source of mass, dV is a volume element and $\frac{\partial }{\partial t}\int_{V}C\left(x,t\right)dV$ is a material time derivative when particle locations are held fixed. Using the Reynolds transport theorem, one can write

(2) $$\frac{\partial }{\partial t}\int_{V}C\left(x,t\right)dV=\int_{V}\left(\frac{\partial C\left(x,t\right)}{\partial t}+\nabla \cdot \left(C\left(x,t\right)v\left(x,t\right)\right)\right)dV\;,$$

where $v\left(x,t\right)$ is the velocity of particles. Followed by localization, Equation (1) gives:

(3) $$\frac{\partial C\left(x,t\right)}{\partial t}+\nabla \cdot \left(C\left(x,t\right)v\left(x,t\right)\right)=\Pi \;.$$

A constitutive statement analogous to Darcy’s law can be proposed for the flux of cells as $q=C\left(x,t\right)v\left(x,t\right)=-k\nabla p\left(x,t\right)$, where $p\left(x,t\right)$ is the pressure inside the tumor and *k* is the proportionality of cell flux to the gradient of pressure, noting that cells move from higher pressure to lower pressure regions. Here we propose that this pressure is linearly proportional to the concentration of cells. Hence, Equation (3) yields:

(4) $$\frac{\partial C\left(x,t\right)}{\partial t}=K\;\nabla^{2}C\left(x,t\right)+\Pi \;,$$

where *K* denotes ability of cells to respond to the gradient of concentration. This property depends on cell diffusivity (*D*) in response to the gradient of the concentration and adhesion forces between cells (*f*), i.e., $K=\hat{K}\left(D,f\right)$. Cell adhesion reduces the ability of cells to move in response to the gradient of concentration. Therefore, to simplify the model we propose a linear relation, K=ϵD, where 0<ϵ<1. The volumetric growth depends on the type of cells, concentrations of nutrients, growth factors and environmental cues. Here we propose a simple linear relation for volumetric growth, $\Pi =\eta \;C\left(x,t\right)$, where η is the rate of cell proliferation. This assumption is correct for early growth of tumor spheroids since they are usually small, such that all cells can receive enough oxygen and nutrient and no hypoxia occurs in the tumor. Under this assumption, Equation (4) can be rewritten as:

(5) $$\frac{\partial C\left(x,t\right)}{\partial t}=K\;\nabla^{2}C\left(x,t\right)+\eta \;C\left(x,t\right)\;.$$

Spherical coordinates, which are well suited to represent the spherical shape of tumor spheroids, can be used to advantage. Since the early stage of growth is radially symmetric, our analysis focuses on radially symmetric solutions. Equation (5) in spherical coordinates become:

(6) $$\frac{\partial C\left(r,t\right)}{\partial t}=\frac{K}{r^{2}}\frac{\partial }{\partial r}\left(r^{2}\frac{\partial C\left(r,t\right)}{\partial r}\right)+\eta \;C\left(r,t\right)\;,$$

subject to the following boundary and initial conditions:

(7a) $$\begin{array}{c} \frac{\partial C\left(r,t\right)}{\partial r}{|}_{r=0}=0\;, \end{array}$$

(7b) $$\begin{array}{c} C\left(r,t\right){|}_{r=R(t)}=C_{0}\;, \end{array}$$

(7c) $$\begin{array}{c} C\left(r,t=0\right)=C_{i}\;,\;\;\mathrm{for}\;\;0<r<R\left(0\right)\;, \end{array}$$

where R(t) is tumor radius, $C_{0}$ is the imposed boundary condition on concentration of cells, and $C_{i}$ is the initial cell concentration, assumed spatially constant. Denoting the free surface boundary by $\Omega \;\left(t\right)=\{x\in R^{3}|\;r-R\left(t\right)=0\}$, the change in radius can be derived by integrating cell motion on the entire volume of the tumor as (see Appendix A.1)

(8) $$\frac{dR\left(t\right)}{dt}=-K\frac{\partial C\left(r,t\right)}{\partial r}{|}_{r=R(t)}\;;\;\;\;R\left(0\right)=R_{0},$$

in which $R_{0}$ is the initial radius. The proliferation inside the tumor increases local concentration of cells and generates a pressure gradient, and consequently a concentration gradient. Equation (5) indicates that cells move from higher concentration regions to lower concentration regions to create a uniform concentration (relaxed concentration) everywhere so that cells do not feel any extra pressure. For this pressure increment to be stabilized by the adhesion forces between cells, the volume of the tumor must increase to reach the relaxed concentration. To model this equilibrium, we introduce boundary condition (7b) which imposes a constant relaxed concentration on the boundary of the tumor spheroid. Solutions to Equations (6) and (8) with corresponding initial and boundary conditions in (7) give the distribution of cells and change in radius of the tumor spheroids over time.

### 2.1. Analytical Solution

Equation (6) is a linear concentration-dependent reaction–diffusion equation with mixed boundary conditions. The reaction–diffusion form with constant source term is:

(9) $$\frac{\partial C\left(r,t\right)}{\partial t}=K\;\nabla^{2}C\left(r,t\right)+\eta C_{i}\;.$$

**Proposition** **1.** *If $C_{1}\left(r,t\right)$ is a solution of* (9), *the following is a solution of* (6) (see Appendix A.2)*:*

(10) $$C\left(r,t\right)=-\eta \int_{0}^{t}C_{1}\left(r,\tau \right)e^{\eta \tau }d\tau +C_{1}\left(r,t\right)e^{\eta t}\;.$$

If a solution to (9) satisfies the initial and boundary conditions (7), then so does (10). Therefore, the first step is to solve Equation (9) subject to initial and boundary conditions (7). Using the variable change $C_{1}\left(r,t\right)=U\left(r,t\right)+C_{0}$, Equation (9) becomes a standard homogeneous PDE with zero boundary conditions and constant initial condition, as follows:

(11a) $$\begin{array}{c} \frac{\partial U\left(r,t\right)}{\partial t}=K\;\nabla^{2}U\left(r,t\right)+\eta C_{i}\;, \end{array}$$

(11b) $$\begin{array}{c} \frac{\partial U\left(r,t\right)}{\partial r}{|}_{r=0}=0\;, \end{array}$$

(11c) $$\begin{array}{c} U\left(R_{1}\left(r\right),t\right)=0\;, \end{array}$$

(11d) $$\begin{array}{c} U\left(r,t=0\right)=C_{i}-C_{0}\;,\;\;\mathrm{for}\;\;0<r<R\left(0\right)\;, \end{array}$$

where $R_{1}\left(t\right)$ is the radius of tumor spheroids without proliferation. The following solution is obtained for $R_{1}\left(t\right)$ (see Appendix A.3):

(12) $$R_{1}\left(t\right)=\frac{C_{i}-C_{0}}{\sqrt{\eta C_{i}}}\;\mathrm{erfi}\sqrt{\eta C_{i}Kt}+R\left(0\right)\;.$$

The solution to the full reaction–diffusion Equation (5), is obtained as (see Appendix A.4):

(13) $$\begin{array}{c} \frac{dR(t)}{dt}=-Kf_{i}(-\eta \sum_{n=1}^{\infty }\frac{{\left(-1\right)}^{n}}{n}\int_{0}^{t}R\left(0\right)e^{(\eta -\lambda_{n}^{2}K+\eta C_{i}K)\tau }F\left(R\left(t\right),n\right)d\tau \\ +R\left(0\right)\sum_{n=1}^{\infty }\frac{{\left(-1\right)}^{n}}{n}e^{(\eta -\lambda_{n}^{2}K+\eta C_{i}K)\tau }F\left(R\left(t\right),n\right))\;. \end{array}$$

### 2.2. Model Simplification

By introducing a new variable, $\bar{R}=n\pi \frac{R(t)}{R_{1}\left(t\right)}$, F(R(t),n) can be written as

(14) $$F\left(R\left(t\right),n\right)=\frac{\bar{R}\left(t\right)\cos\left(\bar{R}\left(t\right)\right)-\sin\left(\bar{R}\left(t\right)\right)}{R{\left(t\right)}^{2}}\;.$$

The timescale of proliferation is small enough compared to that of cell motility such that at each instant the radius of the diffusion-only model, $R_{1}\left(t\right)$, is close to the concentration-dependent reaction–diffusion one, R(t). In the limit of separation of time scales this approximation becomes exact. Therefore, it can be assumed that $\bar{R}\left(t\right)\approx n\pi$. Substitution of Equation (14) into Equation (13) simplifies the rate of change of radius to

(15) $$\frac{dR(t)}{dt}=-\pi Kf_{i}\left(-\eta \int_{0}^{t}\frac{R(0)}{R^{2}\left(\tau \right)}\sum_{n=1}^{\infty }e^{(\eta -\lambda_{n}^{2}K+\eta C_{i}K)\tau }d\tau +\frac{R(0)}{R^{2}\left(t\right)}\sum_{n=1}^{\infty }e^{(\eta -\lambda_{n}^{2}K+\eta C_{i}K)t}\right)\;,$$

which, after simplifications, has the following solution (see Appendix A.5)

(16) $$R\left(t\right)=\alpha \left(\frac{\sqrt{\pi }\;\left(2\eta t-1\right)}{2\sqrt{\eta }}\;\mathrm{erfi}\left(\sqrt{\eta t}\right)-\sqrt{t}\;e^{\eta t}\right)\;.$$

### 2.3. Numerical Solution

In this section, numerical solutions of the model expressed in Equations (6) and (8) are presented. Equation (6) is a reaction–diffusion equation with mixed boundary conditions coupled with the ODE in Equation (8). To solve this system of equations, temporal and spatial discretizations are required. The boundary of the tumor spheroid is moving in time, which requires a new spatial discretization at each time-step. To keep the number of nodes constant, the position of each node must be able to move in time. To this end, a mapping is introduced by non-dimensional variables $\bar{t}=\frac{t}{\tau }$ and $\bar{r}\left(\bar{t}\;\right)=\frac{r}{R\left(\bar{t}\;\right)}$, where $\tau =t_{max}$ and $R(\bar{t})$ is the moving boundary of the tumor. Using this change of variables, the moving domain of the solution is mapped to a new domain which always stays between zero and one. Equations (6) and (8) can be rewritten in the new variables as

(17a) $$\begin{array}{c} \frac{\partial C\left(r,\bar{t}\;\right)}{\partial \bar{t}\;}=\lambda \left(\frac{2}{\bar{r}}\frac{\partial C\left(\bar{r},\bar{t}\;\right)}{\partial \bar{r}}+\frac{\partial^{2}C\left(\bar{r},\bar{t}\;\right)}{\partial {\bar{r}}^{2}}\right)+\eta \;\tau \;C\left(\bar{r},\bar{t}\;\right) \end{array}$$

(17b) $$\begin{array}{c} \frac{dR\left(\bar{t}\right)}{d\bar{t}}=-\lambda R\left(\bar{t}\right)\frac{\partial C\left(\bar{r},\bar{t}\;\right)}{\partial \bar{r}}{|}_{\bar{r}=1}\;, \end{array}$$

where $\lambda =\frac{\tau K}{R{\left(\bar{t}\;\right)}^{2}}$ , subject to the following boundary and initial conditions:

(18a) $$\begin{array}{c} \frac{\partial C\left(\bar{r},\bar{t}\right)}{\partial \bar{r}}{|}_{\bar{r}=0}=0\;, \end{array}$$

(18b) $$\begin{array}{c} C\left(\bar{r},\bar{t}\right){|}_{\bar{r}=1}=C_{0}\;, \end{array}$$

(18c) $$\begin{array}{c} C\left(\bar{r},\bar{t}\right){|}_{\bar{t}=0}=C_{i}\;,\;\;\mathrm{for}\;\;0<\bar{r}<1\;, \end{array}$$

(18d) $$\begin{array}{c} R\left(0\right)=R_{0}\;. \end{array}$$

Here to solve this system of equations, the Crank–Nicolson (CN) finite difference scheme was employed [51] (see Appendix A.6).

## 3. Results and Discussion

### 3.1. Model Analysis

**Figure 1:**
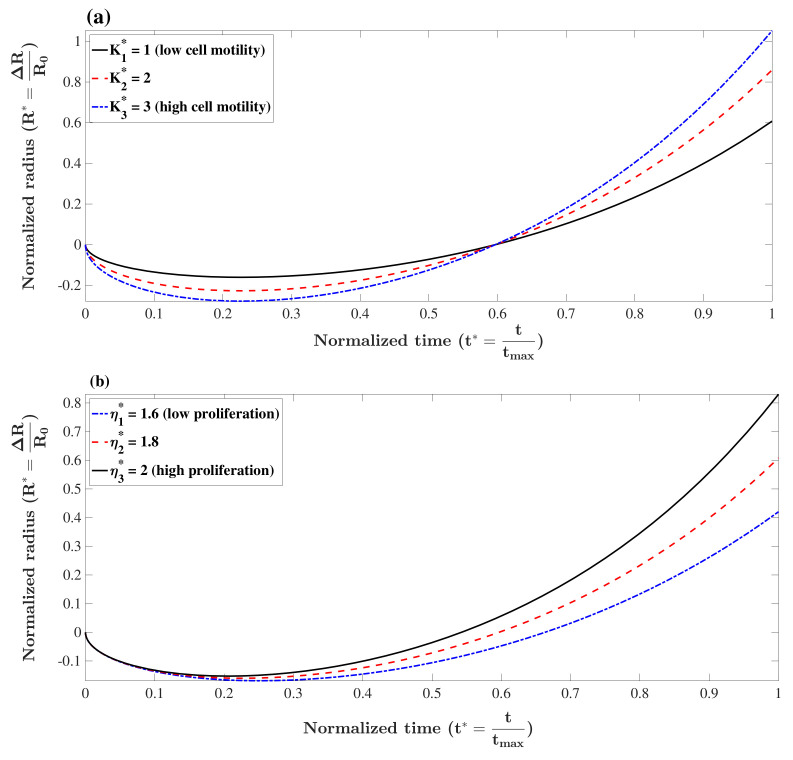
Formation of tumor spheroids with different values of (**a**) cell motility, $K_{1}^{*}=1$, $K_{2}^{*}=2$ and $K_{3}^{*}=3$ (with $\eta^{*}=1.8$, $\frac{C_{0}}{C_{i}}=1.5$ and $R_{0}=0.01$ cm), (**b**) proliferation rate, $\eta_{1}^{*}=1.6$, $\eta_{2}^{*}=1.8$ and $\eta_{3}^{*}=2$ (with $K^{*}=1$, $\frac{C_{0}}{C_{i}}=1.5$ and $R_{0}=0.01$ cm). A tumor spheroid with higher cell motility grows faster since cells can rapidly respond to local proliferation and reach balance by moving the boundary of the tumor. Additionally, a tumor spheroid with a higher proliferation rate assembles faster.

The term α in Equation (16) contains the influence of both cell motility in response to the gradient of concentration, *K*, and the relaxed concentration of cells, $C_{0}$. The higher the absolute difference, $C_{0}-C_{i}$, the more shrinkage is expected. This qualitative effect holds for *K* as well, i.e., the higher the motility, the faster cells respond to the gradient of concentration. To show this effect quantitatively, the value of the normalized radius, $R^{*}\left(t\right)=\frac{R\left(t\right)-R_{0}}{R_{0}}$, versus normalized time, $t^{*}=\frac{t}{t_{max}}$, is plotted in Figure 1a for $C_{i}⩽C_{0}$ and different values of $K^{*}=\frac{K}{K_{0}}$ holding other parameters fixed. Please note that we selected $t_{max}=210$ h to be consistent with the experimental results in the next section. Additionally, we take $K_{0}$ to be $10^{-10}$ cm^2^·s^−1^[52]. As can be seen, the shrinkage of the tumor is faster for cells with higher motility (*K*). The tumor spheroid decays further until the concentration of cells reaches the relaxed concentration where the diffusivity of cells and adhesion forces are in balance (minimum tumor radius). At this point, the proliferation continues to elevate cell concentration and breaks the balance. To reach a new balance, the tumor spheroid increases its radius to reduce the local concentration which finally leads to monotonic growth. Rate of proliferation for highly proliferative cancer cells is normally in the order of $\eta_{0}=10^{-2}\;$ h^−1^. This value was used as a reference number to non-dimensionalize the proliferation rates in our analysis. The effect of cell motility on formation of spheroids is illustrated in Figure 1a in which parameters are set as $\eta =1.8\times \eta_{0}$, $\frac{C_{0}}{C_{i}}=1.5$ and $R_{0}=0.01$ cm. The higher *K* in the figure corresponds to lower minimum radius and faster shrinkage. This result also shows that tumor spheroids with higher *K* grow faster since cells can rapidly respond to local proliferation and reach balance by moving the boundary of the tumor. Unlike many types of mammalian cells which have an intrinsic cell program that restricts their proliferation, most cancer cells are highly proliferative [2]. When cells proliferate, the local concentration increases, and the generated pressure moves cells away. The formation of a tumor spheroid is faster if cells have a high proliferation rate. To illustrate this effect, the formation of a tumor spheroid is depicted in Figure 1b for different values of $\eta^{*}=\frac{\eta }{\eta_{0}}$, holding other parameters fixed, i.e., $K^{*}=1$, $\frac{C_{0}}{C_{i}}=1.5$ and $R_{0}=0.01$ cm. As can be seen, a tumor spheroid with a higher proliferation rate assembles faster.

**Figure 2:**
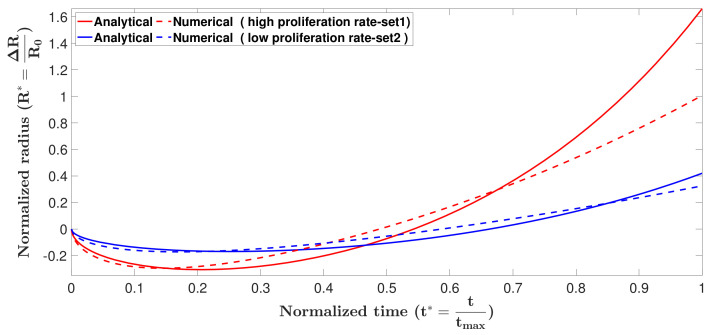
Formation of tumor spheroid obtained by analytical solution and numerical prediction, using two sets of parameters; set 1: $\eta^{*}=1.6,$$\frac{C_{0}}{C_{i}}=1.5,\;K^{*}=1,\;R_{0}=0.01$ cm, and set 2: $\eta^{*}=2,$$\;\frac{C_{0}}{C_{i}}=2,\;K^{*}=1,\;R_{0}=0.02$ cm. In the contraction phase the analytical and numerical solutions reasonably match. The analytical solution loses accuracy once growth becomes dominant. This is a result of the simplification we made in Equation (14).

To compare the analytical and numerical solutions, the formation of the tumor spheroids was obtained for two sets of parameters, (i) $\eta^{*}=1.6$, $\frac{C_{0}}{C_{i}}=1.5$, $K^{*}=1$, $R_{0}=0.01$ cm, and (ii) $\eta^{*}=2$, $\frac{C_{0}}{C_{i}}=2$, $K^{*}=1$, $R_{0}=0.02$ cm, using the Crank–Nicolson scheme outlined in Appendix A.6. Results are compared with the analytical solution in Figure 2.

As can be seen in the figure, analytical and numerical solutions match very well in the contraction phase. The analytical solution loses accuracy once growth becomes dominant. This is a result of the simplification we made in Equation (14). Both solutions predict that the tumor spheroid in parameter set 2, which has higher proliferation rate, experiences faster contraction and faster growth.

### 3.2. Model Validation

**Figure 3:**
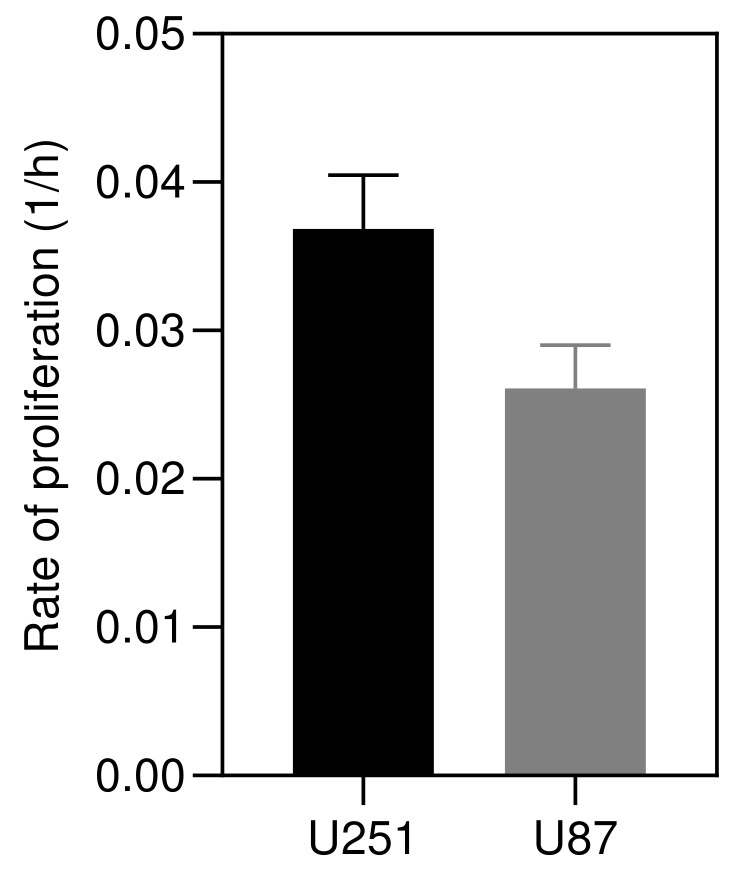
Proliferation rates of U251 and U87 cells cultured in DMEM supplemented with 10% (*v*/*v*) Fetal Bovine Serum (FBS) and 1% (*v*/*v*) Penicillin/Streptomycin, and incubated at 37 °C in 5% CO${}_{2}$. Rates were calculated by counting cells using Trypan blue assay over 24 h (*n* = 3).

**Figure 4:**
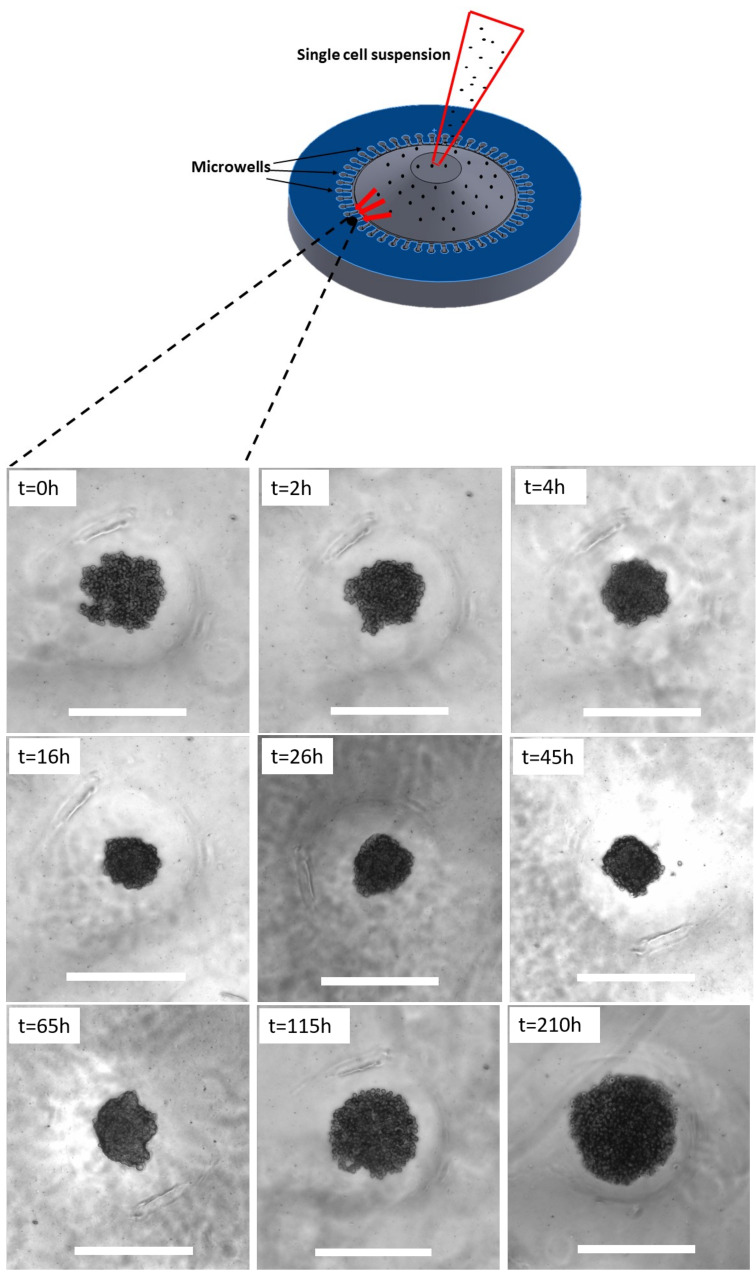
*In vitro* formation of U251- spheroids over 210 h. Spheroids undergo an initial shrinkage and subsequent growth due to the competition between adhesion forces and proliferation pressure. Scale bars show 500μm.

In this section, the theoretical model is validated against the formation of *in vitro* solid tumor spheroids generated from glioma cell lines (U251 and U87 hGB cells), as the most lethal type of intracranial tumors. Reproducibility, ease of assembly and ability to provide high-throughput screening make them a promising candidate for *in vitro* three-dimensional tumor models. Figure 3 shows the rates of proliferation of the two cell lines, $\eta_{U251}=0.037\pm 0.004\;$ h^−1^ and $\eta_{U87}=0.026\pm 0.003\;$ h^−1^, which are in the range of data reported in [53], i.e., $\eta_{U251}=0.038\;$ h^−1^ and $\eta_{U87}=0.033\;$ h^−1^. Results of U251 spheroid formation over 210 h are shown in Figure 4. During the formation phase, intercellular interactions generate adhesion forces which pull cells together and increase the concentration of cells within the spheroid. The size of the spheroid reduces since the proliferation is not yet dominant. The tumor spheroid shrinks until the concentration reaches the relaxed concentration ($C_{0}$). At this minimum radius, the driving forces are in balance, i.e., adhesion forces and forces due to high concentration of cells within the spheroid. This balance breaks once the proliferation of cells becomes dominant, elevating the local concentration above the relaxed concentration. To remove the produced force, the boundary of the tumor spheroid moves to increase the volume. This volume increment reduces the concentration of cells and equilibrates the forces inside the tumor spheroid.

**Figure 5:**
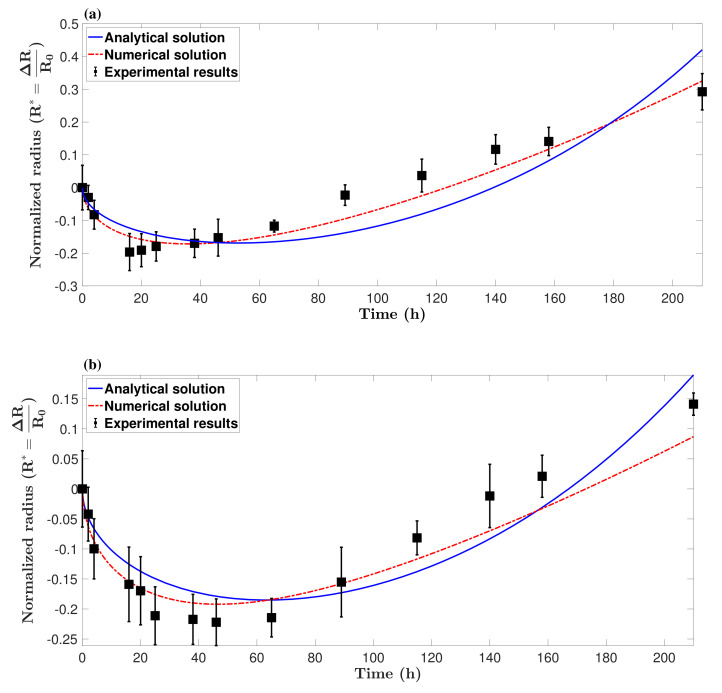
Formation of a tumor spheroid over time, obtained by spheroid culture of hGB cancer cell lines (*n* = 3), (**a**) U251 and (**b**) U87, and compared with both analytical and numerical predictions. The model is able to predict the formation of tumor spheroids and the minimum diameter, but loses accuracy after approximately 160∼180 h. This divergence from experimental results is started when the tumor spheroids lose their homogeneity due to hypoxia and/or necrosis initiation.

For spheroids which did not have a full spherical shape, the average of the largest and smallest diameters was considered to be the spheroid diameter. Figure 5 shows the size of the tumor spheroids over time compared with analytical and numerical solutions. As shown in the figure, the mathematical model provides a reasonable prediction of the formation of tumor spheroids and the minimum diameter. The model predictions were able to follow the trend of formation and growth until approximately 160∼180 h, after which the tumor spheroids lost their homogeneity in terms of cell viability level. It is evident that in big spheroids, cells close to the core become hypoxic and change their metabolism. This can reduce the accuracy of the model.

## 4. Experimental Methodology

### 4.1. Proliferation Rate

HGB cells were cultured in Dulbecco’s Modification of Eagle’s Medium (DMEM) supplemented with 10% (*v*/*v*) Fetal Bovine Serum (FBS) and 1% (*v*/*v*) Penicillin/Streptomycin, and incubated at 37 °C in 5% CO${}_{2}$. The number of cells, $N_{1}$ and $N_{2}$, was counted using a Trypan blue assay after 24 h and 48 h, respectively (fresh media was added after 24 h). Rates of proliferation (1/h) were calculated, shown in Figure 3, for both cell lines as

(19) $$\eta =\frac{N_{2}-N_{1}}{N_{1}*24}.$$

### 4.2. Spheroid Culture

For culturing spheroids, cells cultured in Section 4.1, were dissociated with Gibco^TM^ Trypsin-EDTA (0.5%) and were centrifuged at 300× *g* for 5 min. After removing the supernatant and suspending the cell pellet in 1 mL of medium, the number of cells was counted using a Trypan blue assay. Afterwards, self-filling micro-well arrays (SFMAs) were used to produce uniform tumor spheroids [54]. The desired concentration of cells was loaded dropwise through guiding channels of SFMAs and were gently seeded into the wells. The microwells were kept in an incubator and imaging started 5 h after seeding to let the cells fully settle in the wells. Cells were supplemented with fresh medium every 24 h to maintain the concentration of nutrients. The formation of spheroids was imaged using optical microscopy (Axio Observer, ZEISS, Oberkochen, Germany) over 210 h. The size of spheroids was measured using ImageJ [55].

## 5. Conclusions

In this work, we have presented an analytical solution for the formation of solid tumor spheroids. The process of tumor spheroid formation includes a preliminary contraction phase where adhesion forces densify cell aggregation. This phase proceeds until the cell concentration reaches a threshold, the so-called “relaxed concentration” at equilibrium. Afterwards, cell proliferation raises concentration and produces pressure which breaks the equilibrium. The tumor spheroid evolves in size to compensate for the generated pressure. This transient phase in formation and growth of tumor spheroids was mathematically modeled using a system of coupled PDE and ODE with appropriate boundary and initial conditions. To validate model predictions, human glioblastoma cancer cell lines were spheroid cultured and their size was imaged over 210 h. Results showed that although the model loses accuracy after approximately 160∼180 h, it can nevertheless provide reliable prediction of the size of the spheroids before they become inhomogeneous.It should be noted that this study is limited to the modeling of solid tumor formation with no access to environmental stimuli such as stroma, immune cells, *etc*. However, our approach has the potential to include the inhibitory effect of drugs using an additional reaction–diffusion equation. The effect of a drug on the tumor development may be expected either to simply extend the contraction phase, or to cause a monotonic contraction after the expansion, in either case due to the apoptotic effect of the drug. 

## Figures and Tables

**Figure A1:**
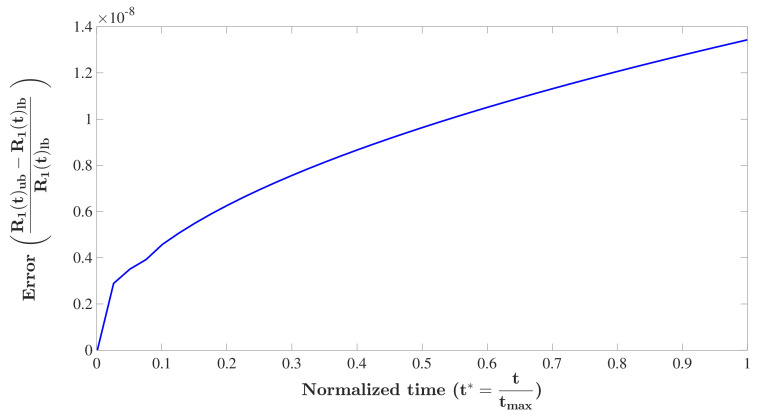
The relative error in the solution of $R_{1}\left(t\right)$ in Equation (A7) using the upper bound, $R_{1}{\left(t\right)}_{ub}$, and lower bound, $R_{1}{\left(t\right)}_{lb}$, of Equation (A9). Parameters are set as $K=10^{-10}$, $R_{0}=0.01$ cm, $t_{max}=$240 h and $\frac{C_{0}}{C_{i}}=2$.

## Appendix A

### Appendix A.1. Rate of Change of Spheroid’s Volume

In this section, we derive an expression to relate the gradient of concentration at the boundary of a tumor to the time rate of change of its radius.

The rate of change of volume is

(A1) $$\frac{dV\left(t\right)}{dt}=\int_{V}\nabla \;\cdot v\left(x,t\right)\;dV=-\int_{V}K\;\nabla^{2}C\left(r,t\right)dV=-\int_{\Omega }K\;\nabla C\left(r,t\right)\cdot n\;d\Omega \;,$$

where n is normal to the surface, i.e., $n={\hat{e}}_{r}$. Additionally, $\nabla C\left(r,t\right)=\frac{\partial C\left(r,t\right)}{\partial r}\;{\hat{e}}_{r}$, and $\frac{dV\left(t\right)}{dt}=4\pi R^{2}\left(t\right)\frac{dR\left(t\right)}{dt}$. The surface element on a sphere of radius *R* is $d\Omega =R^{2}\sin\left(\phi \right)d\theta d\phi$, where θ and ϕ are azimuthal and polar angles, respectively. Hence, Equation (A1) gives:

(A2) $$4\pi R^{2}\left(t\right)\frac{dR\left(t\right)}{dt}=-\int_{0}^{\pi }\int_{0}^{2\pi }K\;\frac{\partial C\left(r,t\right)}{\partial r}{\hat{e}}_{r}\cdot {\hat{e}}_{r}\;R^{2}\left(t\right)\sin\left(\phi \right)\;d\theta d\phi \;,$$

which further simplifies to:

(A3) $$\frac{dR\left(t\right)}{dt}=-K\frac{\partial C\left(r,t\right)}{\partial r}{|}_{r=R(t)}\;.$$

### Appendix A.2. Proof of Proposition 1

In this section, the proof of Proposition 1, which was used as an intermediate step to solve Equation (6), is provided.

Taking derivatives of (10) gives:

(A4a) $$\begin{array}{c} \frac{\partial C\left(r,t\right)}{\partial r}=-\eta \int_{0}^{t}\frac{\partial C_{1}\left(r,t\right)}{\partial r}e^{\eta \tau }d\tau +\frac{\partial C_{1}\left(r,t\right)}{\partial r}e^{\eta t}\;, \end{array}$$

(A4b) $$\begin{array}{c} \frac{\partial^{2}C\left(r,t\right)}{\partial r^{2}}=-\eta \int_{0}^{t}\frac{\partial^{2}C_{1}\left(r,t\right)}{\partial r^{2}}e^{\eta \tau }d\tau +\frac{\partial^{2}C_{1}\left(r,t\right)}{\partial r^{2}}e^{\eta t}\;, \end{array}$$

(A4c) $$\begin{array}{c} \frac{\partial C\left(r,t\right)}{\partial t}=\frac{\partial C_{1}\left(r,t\right)}{\partial t}e^{\eta t}\;. \end{array}$$

By substitution, it can be shown that (A4) satisfies Equation (6), as follows.

$$\frac{\partial C_{1}}{\partial t}=\frac{K}{r^{2}}\frac{\partial }{\partial r}\left(r^{2}\frac{\partial C_{1}}{\partial r}\right)+\eta C_{i}$$

by assumption. The LHS of (6) is

$$\frac{\partial C}{\partial t}=\frac{\partial C_{1}}{\partial t}e^{\eta t}\;.$$

The RHS of (6) is

$$\begin{array}{ccc} \frac{K}{r^{2}}\frac{\partial }{\partial r}\left(r^{2}\frac{\partial C}{\partial r}\right)+\eta \;C & = & \frac{K}{r^{2}}\frac{\partial }{\partial r}\left(r^{2}\left[-\eta \int_{0}^{t}\frac{\partial C_{1}}{\partial r}e^{\eta \tau }d\tau +\frac{\partial C_{1}}{\partial r}e^{\eta t}\right]\right)+\eta C\\ & = & \frac{K}{r^{2}}\frac{\partial }{\partial r}\left(r^{2}\frac{\partial C_{1}}{\partial r}\right)e^{\eta t}-\eta \int_{0}^{t}\frac{K}{r^{2}}\frac{\partial }{\partial r}\left(r^{2}\frac{\partial C_{1}}{\partial r}\right)e^{\eta \tau }d\tau +\eta C\\ & = & \left(\frac{\partial C_{1}}{\partial t}-\eta C_{i}\right)e^{\eta t}-\eta \int_{0}^{t}\left(\frac{\partial C_{1}}{\partial \tau }-\eta C_{i}\right)e^{\eta \tau }d\tau +\eta C\\ & = & \frac{\partial C_{1}}{\partial t}e^{\eta t}-\eta C_{i}e^{\eta t}-\eta \left[{\left.C_{1}e^{\eta \tau }\right|}_{0}^{t}-\eta \int_{0}^{t}C_{1}e^{\eta \tau }d\tau \right]+\eta C_{i}\int_{0}^{t}\eta e^{\eta \tau }d\tau +\eta C\\ & = & \frac{\partial C_{1}}{\partial t}e^{\eta t}-\eta C_{i}e^{\eta t}-\eta \left[C-C_{i}\right]+\eta C_{i}\left(e^{\eta t}-1\right)+\eta C\\ & = & \frac{\partial C_{1}}{\partial t}e^{\eta t}\;. \end{array}$$

Hence Equation (6) is satisfied.

### Appendix A.3. Solution of R_*1*_ (t)

Here, we solve Equation (11a) and derive an approximate expression for $R_{1}\left(t\right)$.

Using separation of variables, $U\left(r,t\right)=P\left(r\right)\;T\left(t\right)$, one can derive the solution of (11a) as

(A5) $$U\left(r,t\right)=\sum_{n=1}^{\infty }D_{n}e^{-(\lambda_{n}^{2}-\eta C_{i})Kt}\;\;\frac{\sin\left(\lambda_{n}r\right)}{r}\;,$$

where $D_{n}=2R\left(0\right)\;\left(C_{0}-C_{i}\right)\frac{{\left(-1\right)}^{n}}{n\pi }$ and $\lambda_{n}=\frac{n\pi }{R_{1}\left(t\right)}$. Hence,

(A6) $$C_{1}\left(r,t\right)=\sum_{n=1}^{\infty }D_{n}e^{-(\lambda_{n}^{2}-\eta C_{i})Kt}\;\;\frac{\sin\left(\lambda_{n}r\right)}{r}+C_{0}\;.$$

Substitution of (A6) into (A3) gives

(A7) $$\frac{dR_{1}\left(t\right)}{dt}=\frac{2K(C_{i}-C_{0})}{R_{1}\left(t\right)}\sum_{n=1}^{\infty }e^{-(\lambda_{n}^{2}-\eta C_{i})Kt}\;.$$

The infinite series in Equation (A7) is convergent and has upper and lower bounds as follows:

(A8) $$\int_{1}^{\infty }e^{-(\lambda_{n}^{2}-\eta C_{i})Kt}dn⩽\sum_{n=1}^{\infty }e^{-(\lambda_{n}^{2}-\eta C_{i})Kt}⩽\int_{0}^{\infty }e^{-(\lambda_{n}^{2}-\eta C_{i})Kt}dn\;.$$

Using the identity $\int e^{-(\lambda_{n}^{2}-\eta C_{i})Kt}dn=\frac{R_{1}\left(t\right)\;e^{\eta C_{i}Kt}}{2\sqrt{\pi Kt}}\;\mathrm{erf}\left(\frac{n\pi \sqrt{Kt}}{R_{1}\left(t\right)}\right)+\mathrm{constant}$, the inequalities in (A8) become

(A9) $$\begin{array}{c} \frac{\sqrt{K}\left(C_{i}-C_{0}\right)}{\sqrt{\pi }}e^{\eta C_{i}Kt}\left(\frac{1-\mathrm{erf}\left(\frac{\pi \sqrt{Kt}}{R_{1}\left(t\right)}\right)}{\sqrt{t}}\right)⩽\frac{2K(C_{i}-C_{0})}{R_{1}\left(t\right)}\sum_{n=1}^{\infty }e^{-(\lambda_{n}^{2}-\eta C_{i})Kt}\\ ⩽\frac{\sqrt{K}\left(C_{i}-C_{0}\right)}{\sqrt{\pi t}}e^{\eta C_{i}Kt}\;. \end{array}$$

Here, we observe that the upper and lower bounds in Equation (A9) are very tight, at least for system parameters in a reasonable range. Therefore, considering either bound as an approximate solution for $\frac{dR_{1}\left(t\right)}{dt}$ is acceptable. To show this, the solution of $R_{1}\left(t\right)$ using the upper bound, $R_{1}{\left(t\right)}_{ub}$, and lower bound, $R_{1}{\left(t\right)}_{lb}$, are found by a finite difference method and the corresponding relative error, $\frac{R_{1}{\left(t\right)}_{ub}-R_{1}{\left(t\right)}_{lb}}{R_{1}{\left(t\right)}_{lb}}$, is shown in Figure A1. System parameters are adopted in the range of biological properties of tumor spheroids as $K=10^{-10}$ cm^2^/s, $R_{0}=0.01$ cm. We also assume that the initial phase of growth does not take longer than a few days, $t_{max}=$240 h, and $\frac{C_{0}}{C_{i}}=2$.

As can be seen in the figure, the error is very small, indicating that both bounds are acceptable in this range. To reduce the complexity in the analytical solution procedure, we used the upper bound as the solution of $\frac{dR_{1}\left(t\right)}{dt}$, for which the analytical solution is available as

(A10) $$R_{1}\left(t\right)=\frac{C_{i}-C_{0}}{\sqrt{\eta C_{i}}}\;\mathrm{erfi}\sqrt{\eta C_{i}Kt}+R\left(0\right)\;.$$

### Appendix A.4. Solution of Full RD Equation

In this section, the solution to the full reaction–diffusion Equation (5) is obtained. By substitution of Equation (A6) into (10), one can obtain:

(A11) $$\begin{array}{c} C\left(r,t\right)=-\eta f_{i}R\left(0\right)\sum_{n=1}^{\infty }\frac{{\left(-1\right)}^{n}}{n}\int_{0}^{t}e^{-(\lambda_{n}^{2}-\eta C_{i})K\tau }\;\;\frac{\sin(\lambda_{n}r)}{r}\;e^{\eta \tau }d\tau \;-\eta C_{0}\int_{0}^{t}e^{\eta \tau }d\tau \\ +R\left(0\right)f_{i}\;\sum_{n=1}^{\infty }\frac{{\left(-1\right)}^{n}}{n}e^{-(\lambda_{n}^{2}-\eta C_{i})Kt}\;\;\frac{\sin(\lambda_{n}r)}{r}e^{\eta t}+C_{0}\;e^{\eta t}\;, \end{array}$$

which further simplifies to:

(A12) $$\begin{array}{c} C\left(r,t\right)=-\eta f_{i}R\left(0\right)\sum_{n=1}^{\infty }\frac{{\left(-1\right)}^{n}}{n}\int_{0}^{t}e^{-(\lambda_{n}^{2}-\eta C_{i})K\tau }\;\;\frac{\sin(\lambda_{n}r)}{r}\;e^{\eta \tau }d\tau \;\\ +R\left(0\right)f_{i}\;\sum_{n=1}^{\infty }\frac{{\left(-1\right)}^{n}}{n}e^{-(\lambda_{n}^{2}-\eta C_{i})Kt}\;\;\frac{\sin(\lambda_{n}r)}{r}e^{\eta t}+C_{0}\;, \end{array}$$

where $f_{i}=\frac{2(C_{0}-C_{i})}{\pi }$. It should be noted that there are two radii in this solution, i.e., $R_{1}\left(t\right)$ and R(t). The former is the radius of the reaction–diffusion form with constant source, Equation (9), and the latter is the radius of the concentration-dependent reaction–diffusion form, Equation (5). By substitution of (A12) into (A3), the first order differential equation for R(t) is obtained as:

(A13) $$\begin{array}{c} \frac{dR(t)}{dt}=-Kf_{i}(-\eta \sum_{n=1}^{\infty }\frac{{\left(-1\right)}^{n}}{n}\int_{0}^{t}R\left(0\right)e^{(\eta -\lambda_{n}^{2}K+\eta C_{i}K)\tau }F\left(R\left(t\right),n\right)d\tau \\ +R\left(0\right)\sum_{n=1}^{\infty }\frac{{\left(-1\right)}^{n}}{n}e^{(\eta -\lambda_{n}^{2}K+\eta C_{i}K)\tau }F\left(R\left(t\right),n\right))\;, \end{array}$$

where $F\left(R,n\right)=\frac{\lambda_{n}R\left(t\right)\cos\left(\lambda_{n}R\left(t\right)\right)-\sin\left(\lambda_{n}R\left(t\right)\right)}{R{\left(t\right)}^{2}}$.

### Appendix A.5. Model Simplification

Here, steps to simplify Equation (15) are provided. Equation (15) gives the rate of change of radius as

(A14) $$\frac{dR(t)}{dt}=-\pi Kf_{i}\left(-\eta \int_{0}^{t}\frac{R(0)}{R^{2}\left(\tau \right)}\sum_{n=1}^{\infty }e^{(\eta -\lambda_{n}^{2}K+\eta C_{i}K)\tau }d\tau +\frac{R(0)}{R^{2}\left(t\right)}\sum_{n=1}^{\infty }e^{(\eta -\lambda_{n}^{2}K+\eta C_{i}K)t}\right)\;.$$

Using the same identity used in (A9) and the argument proposed in deriving Equation (A10), Equation (A14) simplifies to

(A15) $$\frac{dR(t)}{dt}=-\alpha \left(-\eta \int_{0}^{t}\frac{R\left(0\right)R_{1}\left(\tau \right)}{R^{2}\left(\tau \right)}\frac{e^{\eta \tau (1+C_{i}K)}}{\sqrt{\tau }}d\tau +\frac{R\left(0\right)R_{1}\left(t\right)}{R^{2}\left(t\right)}\frac{e^{\eta t(1+C_{i}K)}}{\sqrt{t}}\right)\;,$$

where $\alpha =\frac{f_{i}\sqrt{\pi K}}{2}$. It should be noted that $C_{i}K\ll 1$, ($1+C_{i}K\approx 1$). Hence, Equation (A15) simplifies approximately to the following nonlinear integro-differential equation:

(A16) $$\frac{dR(t)}{dt}=-\alpha \left(-\eta \int_{0}^{t}\frac{R\left(0\right)R_{1}\left(\tau \right)}{R^{2}\left(\tau \right)}\frac{e^{\eta \tau }}{\sqrt{\tau }}d\tau +\frac{R\left(0\right)R_{1}\left(t\right)}{R^{2}\left(t\right)}\frac{e^{\eta t}}{\sqrt{t}}\right)\;.$$

Please note that R(0) is the initial radius, and $R_{1}\left(t\right)$ and R(t) are radii for the constant-source reaction–diffusion equation and the concentration-dependent reaction–diffusion equation, respectively. It can be shown that the term $\frac{R\left(0\right)R_{1}\left(t\right)}{R^{2}\left(t\right)}$ fluctuates around 1 during the shrinkage and early growth phase as follows:

(A17a) $$\begin{array}{c} \frac{R\left(0\right)R_{1}\left(t\right)}{R^{2}\left(t\right)}=1,\;\;\;\mathrm{at}\;t=0\;, \end{array}$$

(A17b) $$\begin{array}{c} \frac{R\left(0\right)R_{1}\left(t\right)}{R^{2}\left(t\right)}>1,\;\;\;\mathrm{in}\;\mathrm{the}\;\mathrm{shrinkage}\;\mathrm{phase}\;, \end{array}$$

(A17c) $$\begin{array}{c} \frac{R\left(0\right)R_{1}\left(t\right)}{R^{2}\left(t\right)}\approx 1,\;\;\;\mathrm{in}\;\mathrm{the}\;\mathrm{transition}\;\mathrm{phase}\;, \end{array}$$

(A17d) $$\begin{array}{c} \frac{R\left(0\right)R_{1}\left(t\right)}{R^{2}\left(t\right)}<1,\;\;\;\mathrm{in}\;\mathrm{the}\;\mathrm{initial}\;\mathrm{growth}\;\mathrm{phase}\;. \end{array}$$

Thus, we write $\frac{R\left(0\right)R_{1}\left(t\right)}{R^{2}\left(t\right)}=1+\varepsilon \left(t\right)$, where ε(t) denotes the variation around 1 and depends on the intrinsic properties of the system, such as proliferation rate (η), cell diffusivity (D), etc. Although the form of function ε(t) cannot be specified precisely, we make the approximation ε(t)≈0 to simplify Equation (A16). This assumption is not accurate, especially for the growth phase. However, the comparison between the analytical solution and both numerical and experimental results in Section 3.1 and Section 3.2 confirms that this approximation provides an acceptable prediction on the formation of spheroids in the range of interest. Using the identity $\int_{0}^{t}\frac{e^{\eta \tau }}{\sqrt{\tau }}d\tau =\sqrt{\frac{\pi }{\eta }}\;\;\;\mathrm{erfi}\left(\sqrt{\eta t}\right)$, Equation (A16) then yields:

(A18) $$\frac{dR(t)}{dt}=-\alpha \left(-\sqrt{\pi \eta }\;\;\mathrm{erfi}\;\left(\sqrt{\eta t}\right)+\frac{e^{\eta t}}{\sqrt{t}}\right)\;;\;R\left(t\right){|}_{t=0}=R_{0}\;.$$

Please note that if $C_{i}=C_{0}$ then α=0, and the size of the tumor does not change, but experimentally, the initial seeding concentration, $C_{i}$, is always less than $C_{0}$, since a compact tumor mass has not yet formed, so this can be considered physically implausible. Using the fact that

(A19) $$\int_{0}^{t}\mathrm{erfi}\left(\sqrt{\eta \tau }\right)d\tau =\frac{\left(2\eta t+1\right)\mathrm{erfi}\left(\sqrt{\eta t}\right)-\frac{2e^{\eta t}\sqrt{\eta t}}{\sqrt{\pi }}}{2\eta }\;,$$

the solution to (A18) can be obtained as

(A20) $$R\left(t\right)=\alpha \left(\frac{\sqrt{\pi }\;\left(2\eta t-1\right)}{2\sqrt{\eta }}\;\mathrm{erfi}\left(\sqrt{\eta t}\right)-\sqrt{t}\;e^{\eta t}\right)\;.$$

### Appendix A.6. Numerical Method

The Crank–Nicolson scheme is implicit, unconditionally stable, and gives second-order convergence. Considering temporal (i) and spatial (j) discretizations, the following approximations to temporal and spatial derivatives transform the differential Equations (6) and (8) into an algebraic system of equations.

(A21a) $$\begin{array}{c} \frac{\partial C(i,j)}{\partial \bar{t}}=\frac{C_{j}^{i+1}-C_{j}^{i}}{\Delta \bar{t}}\;, \end{array}$$

(A21b) $$\begin{array}{c} \frac{dR(i)}{d\bar{t}}=\frac{R^{i+1}-R^{i}}{\Delta \bar{t}}\;, \end{array}$$

(A21c) $$\begin{array}{c} \frac{\partial C(i,j)}{\partial \bar{r}}=\frac{1}{2}\left(\frac{C_{j+1}^{i}-C_{j-1}^{i}}{\Delta \bar{r}}+\frac{C_{j+1}^{i}-C_{j-1}^{i}}{\Delta \bar{r}}\right)\;, \end{array}$$

(A21d) $$\begin{array}{c} \frac{\partial^{2}C\left(i,j\right)}{\partial {\bar{r}}^{2}}=\frac{1}{2}\left(\frac{C_{j+1}^{i+1}-2C_{j}^{i+1}+C_{j-1}^{i+1}}{\Delta {\bar{r}}^{2}}+\frac{C_{j+1}^{i}-2C_{j}^{i}+C_{j-1}^{i}}{\Delta {\bar{r}}^{2}}\right)\;. \end{array}$$

Substitution of these approximations into (17) gives a system of linear algebraic equations in the form of Ax=b, where A is square matrix of coefficients and vector x contains unknown concentrations at each node. The CN scheme is implicit in both time and space. Based on this method, the ${\left(k+1\right)}^{th}$ iteration of unknown concentrations, $C^{\left(k+1\right)}$, is defined as:

(A22) $$C^{\left(k+1\right)}=A_{lower}^{-1}\left(b-A_{upper}C^{\left(k\right)}\right),$$

where $A_{lower}$ and $A_{upper}$ are respectively a lower triangular and strictly upper triangular decomposition of matrix *A*. As the iterations proceed, the approximations converge until the error reaches a defined tolerance. MATLAB^®^ [56] was used to perform iterations to obtain unknown concentrations at each node. Please note that we used explicit value of R(t) in Equation (17a), i.e., $R^{i}\left(t\right)$, such that we could solve for the concentrations first and plug them into Equation (17b) to obtain $R^{i+1}\left(t\right)$.
